# DTI-based upper limit of voxel free water fraction

**DOI:** 10.1016/j.heliyon.2018.e00700

**Published:** 2018-07-20

**Authors:** Paul M. Macey, M. Albert Thomas, Luke A. Henderson

**Affiliations:** a UCLA School of Nursing, University of California at Los Angeles, Los Angeles, California, USA; b Brain Research Institute, David Geffen School of Medicine at UCLA, University of California at Los Angeles, Los Angeles, California, USA; c Department of Radiological Sciences, David Geffen School of Medicine at UCLA, University of California at Los Angeles, Los Angeles, California, USA; d Department of Anatomy, University of Sydney, Sydney, New South Wales, Australia

**Keywords:** Medical imaging, Neuroscience

## Abstract

**Background:**

Free water (FW) in neuroimaging is non-flowing extracellular water in the cranium and brain tissue, and includes both cerebral spinal fluid (CSF) and fluid in intercellular space or edema. For a region such as a voxel (spatial unit of measurement in neuroimaging), the FW fraction is defined as the volume fraction of FW within that volume. Quantifying the FW fraction allows estimating contamination by fluid of neuroimaging or magnetic resonance spectroscopy measurements within a voxel.

**New method:**

An upper limit to the fraction of FW within a voxel, based on any diffusion tensor imaging (DTI) sequence including a standard single shell at one b-value, can be derived from the standard diffusion tensor by scaling the third eigenvalue of the diffusion tensor. Assuming a two-compartment model, the diffusivity of a voxel is a combination of tissue and FW diffusivity. FW fraction is FW volume divided by voxel volume. Assuming FW diffuses equally in all directions, the diffusivity component is representable by a single, non-tensor diffusivity value. Since the diffusivity of water is known for a given temperature, and brain temperature is relatively constant, the FW diffusivity value can be assumed constant. The third eigenvector of the voxel diffusion tensor is the direction of least diffusivity and since the FW component of diffusivity is equal in all directions, we show that FW diffusivity cannot be lower than the third eigenvalue. Assuming FW contributes proportionally to voxel diffusivity, we show that the third eigenvalue divided by water diffusivity (as a constant based on known water diffusivity at 36.7 °C) forms an upper limit on the FW-fraction (*f*_*UL*_).

**Results:**

We calculated *f*_*UL*_ for 384 subjects from the IXI dataset. Values mostly ranged from 0.1 to 0.6, and were closely related to radial diffusivity.

**Comparison with Existing Methods:***f*_*UL*_ is easily calculated from any DTI data, but is not a true estimate of FW-fraction.

**Conclusions:**

The *f*_*UL*_ measure offers a starting point in calculating the true FW-fraction of a voxel, or an easy-to-calculate voxel characteristic.

## 1 Introduction

Free water in the context of neuroimaging refers to extracellular non-flowing water in the cranium and brain tissue. Quantifying free water is of interest for estimating cerebral spinal fluid (CSF) contamination and as an index measured within brain tissue since it is sensitive to edema from pathologies. Free water is characterized by uninhibited movement, and is present in CSF and vasogenic edema. A conceptual approach to measuring free water is using diffusion tensor imaging (DTI) estimates of water diffusivity to classify free water regions based on high diffusivity. If DTI scans had sub-nanometer resolution, one could measure free water directly, since most voxels could be categorized as being either water or tissue. However, at the current practical voxel resolution of 1–3 mm in each dimension, a DTI voxel encompasses millions of cells and intercellular space, and will often include brain-CSF boundaries. As a consequence, many voxels will present with partial volume effects, and measures of diffusivity in a voxel reflect a contributions from tissue and free water. The aim of DTI studies is usually to assess tissue structure, but contamination from CSF and edema affect mean diffusivity and factional anisotropy values [1, 2]. Identifying and accounting for potential free-water contamination could lead to better measures of tissue structure and higher fraction of intracellular water. An estimate of free water from DTI requires further calculations than standard processing based on a voxel model comprising free water and tissue, termed a two-compartment model [3]. Existing methods have been proposed to estimate the proportion of free water within a voxel, termed the free water fraction *f*, based on the ratio of the volume of free water *V*_*fw*_ to the volume of the voxel *V*_*vox*_ (1):

(1) $$f=\frac{V_{fw}}{V_{vox}}\text{.}$$

However, these methods may not have single solutions [4, code no longer available], leading to the need to solve an inverse problem with optimization, and approaches may require the addition of non-standard DTI scans with specific parameters [5, 6, 7]. We therefore propose a method to provide an estimate related to free water content. Specifically, we present a calculation, based on any product DTI sequence, which provides an upper bound to the free water fraction.

As background, the motivation to measure free water in the context of assessing CSF contamination is present in several imaging modalities. For example, magnetic resonance spectroscopy (MRS) involves large voxels (X/Y/Z dimensions of 5–15 mm) with large partial volume effects, and MRS measures are highly influenced by the proportions of tissue and free water in the voxel [8]. Neurochemical levels are usually presented as a ratio of creatine, which is used as a marker of tissue cell content [9, 10], but the free water fraction would provide additional information, especially in the context of diseases that alter creatine levels or chemicals in CSF. Theoretically, free water fraction could help with interpretation of structural measures such as T2 relaxometry, voxel-based morphometry, cerebral blood flow with arterial spin labelling, and functional MRI. DTI itself involves measuring a tensor based on the directionality of water diffusion properties, and even at ∼1 mm resolution, DTI voxels will have partial CSF components. Therefore, by extracting the free water fraction, improved estimates of the tissue-specific tensor are possible [4]. A two-compartment model was shown to allow for better estimation of tissue tensors [3].

Another motivation to measure free water fraction is its sensitivity to changes in intracellular water and space that occur in pathologies associated with edema, or inflammation, as well as atrophy [11]. Initial studies have found changes in free water associated with aging [12], and diseases such as schizophrenia [13], multiple-sclerosis [11], and Alzheimer's disease [5]. An estimate related to free water would be another quantitative MRI measure with which to assess neural pathology, or provide a starting point for free water calculation by another method.

## 2 Theory

### 2.1 Two-compartment model

**Fig. 1:**
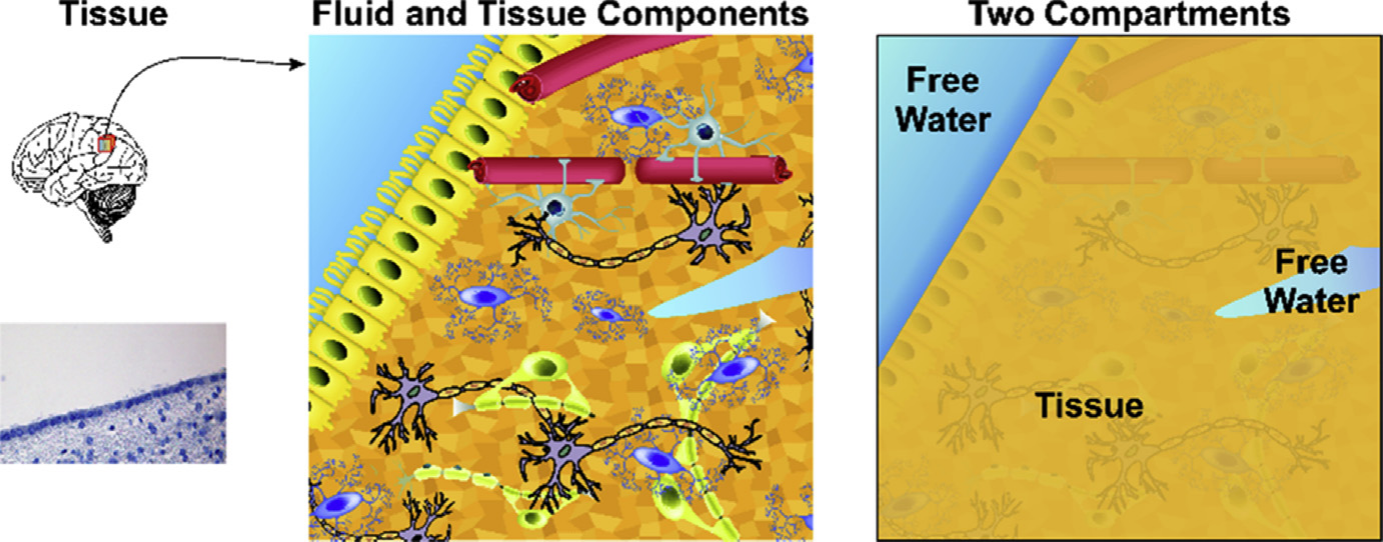
Two-compartment model based on tissue and fluid in brain. Left panel shows image from animal brain of boundary between brain tissue and CSF. Middle panel illustrates various tissue and fluid components, which are simplified into the two-compartment model in the right panel. Note that the free water compartment includes CSF and water in the tissue.

Considering the underlying structure of the brain, two-compartment models have been proposed, whereby the characteristics of a voxel tensor reflect the combination of components from different types of material [3]. Fig. 1 illustrates this model in a hypothetical voxel, illustrating a brain-CSF boundary in an animal model (Fig. 1, left panel). A voxel may include CSF and brain tissue, and within brain tissue there may be edema, in addition to neurons and glial cells, vessels, and other intercellular material or ependymal cells (Fig. 1, middle panel). Free water exists both in CSF and throughout the tissue, but for the purposes of the model, we assume one compartment is free water and the other brain tissue (Fig. 1, right panel).

Standard DTI measures are interpreted assuming a single compartment model. The conventional DTI-derived diffusion tensor ***D*** represents the directional diffusivity of water within a voxel [14], and this tensor forms the basis of most DTI analyses, including calculation of structural indices and fiber or probabilistic tracking. The most common formulation is a 3×3 matrix that is assumed to be symmetric (2):

(2) $$D=\left[\begin{array}{ccc} D_{xx} & D_{xy} & D_{xz}\\ D_{xy} & D_{yy} & D_{yz}\\ D_{xz} & D_{yz} & D_{zz} \end{array}\right]$$

For a two compartment model, the diffusivity of a voxel ***D***_***vox***_ is the combination of diffusivities from tissue (***D***_***t***_) and free water (***D***_***fw***_) compartments (3):

(3) $$D_{\mathrm{vox}}=\;\left(1-f\right)D_{t}+fD_{\mathrm{fw}},$$

assuming the voxel diffusivity is a linear function of compartment diffusivities [15]. Assuming the free water diffusion is equal in all directions [4], the tensor ***D***_***fw***_ water simplifies to (4):

(4) $$D_{\mathrm{fw}}=\left[\begin{array}{ccc} {\text{D}}_{\text{W}} & 0 & 0\\ 0 & {\text{D}}_{\text{W}} & 0\\ 0 & 0 & {\text{D}}_{\text{W}} \end{array}\right]$$

where ${\text{D}}_{\text{W}}$ is the diffusivity of water.

While (2) can be solved using any DTI series with 6 or more directions, for Eq. (3), even though only one additional variable is introduced, *f* does not have a single solution and is complex to solve for. Here, we propose using a characteristic of the diffusion tensor to obtain an estimate of an upper limit of the free water compartment fraction.

### 2.2 Third eigenvalue of the diffusion tensor and free water fraction

**Fig. 2:**
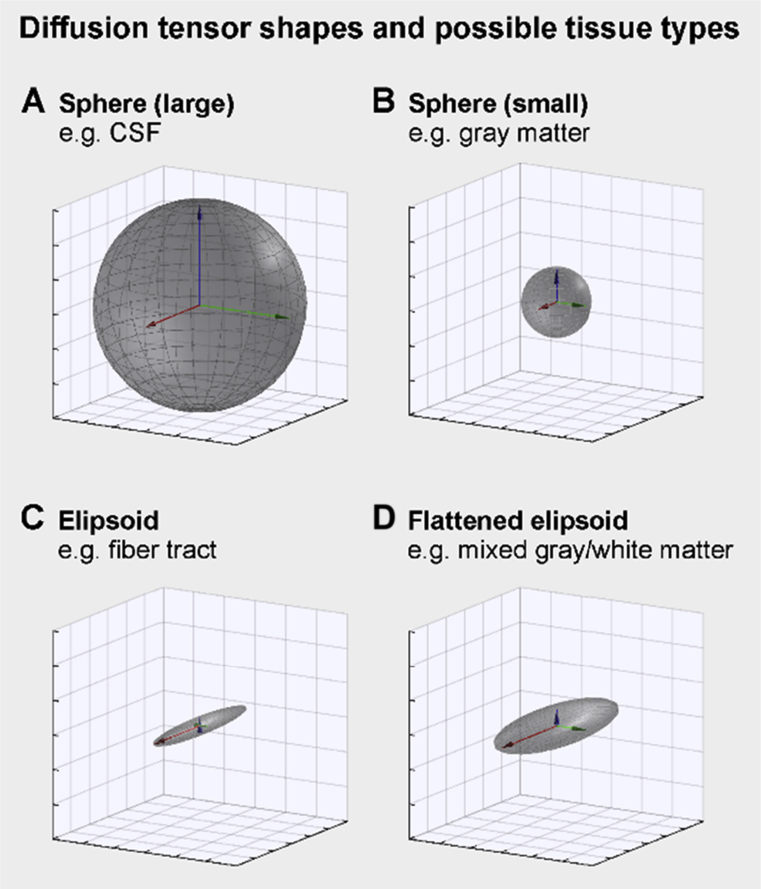
Illustration of different shapes of diffusion tensor and possible corresponding tissue types. Considering the eigenvalues *λ*_*1-3*_ of the tensor, the smallest eigenvalue relates to the others as follows: A) *λ*_*3*_ = *λ*_*2*_ = *λ*_*1*_; B) *λ*_*3*_ = *λ*_*2*_ = *λ*_*1*_; C) *λ*_*3*_ = *λ*_*2*_ < *λ*_*1*_; and D) *λ*_*3*_ < *λ*_*2*_ < *λ*_*1*_.

The tensor ***D*** can be decomposed into eigenvectors, with the first eigenvector *v*_*1*_ being in the direction of greatest diffusivity. The second eigenvector *v*_*2*_ is the direction of greatest diffusivity in a plane perpendicular to the first eigenvector, and the third *v*_*3*_ is in the orthogonal direction of least diffusivity. The three eigenvalues, *λ*_*1*_, *λ*_*2*_, *λ*_*3*_, represent the magnitude of diffusivity along the three eigenvectors. The diffusion tensor can be visualized by an ellipsoid, as in Fig. 2. In fluid such as CSF, water can diffuse unimpeded by barriers equally in all directions, and so the mean and directional diffusivities will be equivalent in magnitude, and approximately equal to the diffusivity of water (Fig. 2A). In brain tissue with minimal directionality in structure, such as some gray matter regions, the diffusion eigenvectors will be of similar magnitude in all directions but the eigenvalues will be much lower than those in CSF [Fig. 2B; 14]. In tissue that is highly directional, such as large white matter tracks like the corpus callosum, one direction will dominate, and the eigenvalue *λ*_*1*_ in that direction will be moderate to large combined with much smaller values for *λ*_*2*_ and *λ*_*3*_ (Fig. 2C). Another scenario is tissue where water can move easily on one direction, and within a plane perpendicular to the first direction, with minimal diffusivity perpendicular to the plane, as illustrated in Fig. 2D. In this scenario, *λ*_*2*_ and *λ*_*3*_ are similar and *λ*_*3*_ is smaller.

**Fig. 3:**
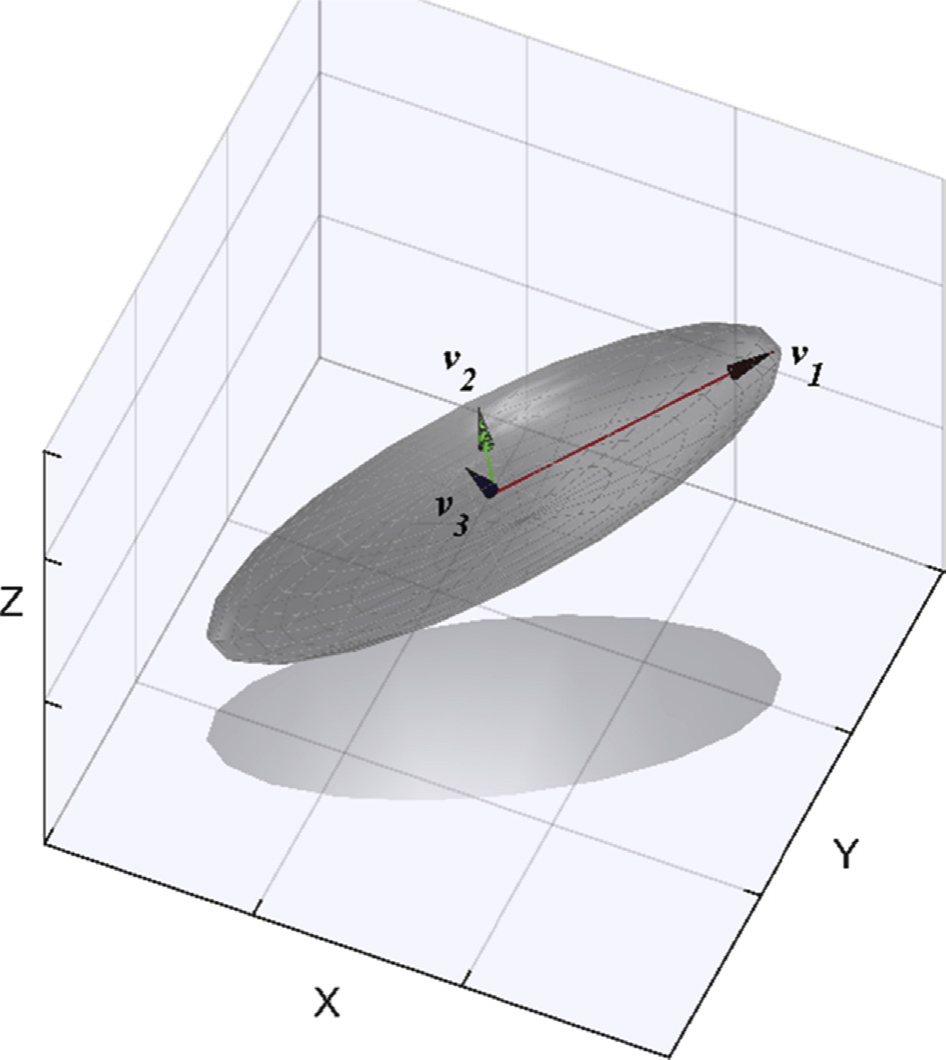
Ellipsoid in rotated Eigenvector coordinate system.

For the purposes of assessing free water, direction is not required, and we can therefore perform calculations on a rotated coordinate system. If we choose the eigenvectors (*v*_*1*_, *v*_*2*_, *v*_*3*_) as the coordinate system (Fig. 3), the rotated voxel diffusion matrix $\Lambda_{\mathrm{vox}}$ is comprised of the eigenvalues along the diagonal (5):

(5) $$\Lambda_{\mathrm{vox}}=\left[\begin{array}{ccc} \lambda_{1} & 0 & 0\\ 0 & \lambda_{2} & 0\\ 0 & 0 & \lambda_{3} \end{array}\right]$$

The two-compartment model in (3) can be formulated in the rotated space, where $\Lambda_{t}$ is the rotated tissue diffusion tensor, as (6):

(6) $$\Lambda_{\mathrm{vox}}=\;\left(1-f\right)\Lambda_{t}+f\Lambda_{\mathrm{fw}}.$$

Since free water is assumed to diffuse without directionality, $\Lambda_{\mathrm{fw}}=\;D_{\mathrm{fw}}$, leading to (7):

(7) $$\Lambda_{\mathrm{vox}}=\left[\begin{array}{ccc} \lambda_{1} & 0 & 0\\ 0 & \lambda_{2} & 0\\ 0 & 0 & \lambda_{3} \end{array}\right]=\left(1-f\right)\Lambda_{t}+f\;\left[\begin{array}{ccc} {\text{D}}_{\text{W}} & 0 & 0\\ 0 & {\text{D}}_{\text{W}} & 0\\ 0 & 0 & {\text{D}}_{\text{W}} \end{array}\right]\text{.}$$

Considering the components of $\Lambda_{\mathrm{vox}}$, we have three linear equations that include *f* (8):

(8) $$\left[\begin{array}{ccc} \lambda_{1} & 0 & 0\\ 0 & \lambda_{2} & 0\\ 0 & 0 & \lambda_{3} \end{array}\right]=\left(1-f\right)\left[\begin{array}{ccc} \Lambda_{11} & \Lambda_{12} & \Lambda_{13}\\ \Lambda_{21} & \Lambda_{22} & \Lambda_{23}\\ \Lambda_{31} & \Lambda_{32} & \Lambda_{33} \end{array}\right]+f\left[\begin{array}{ccc} {\text{D}}_{\text{W}} & 0 & 0\\ 0 & {\text{D}}_{\text{W}} & 0\\ 0 & 0 & {\text{D}}_{\text{W}} \end{array}\right]\Rightarrow \lambda_{1}=\left(1-f\right)\Lambda_{11}+{\text{fD}}_{\text{W}}\lambda_{2}=\left(1-f\right)\Lambda_{22}+f{\text{D}}_{\text{W}}\lambda_{3}=\left(1-f\right)\Lambda_{33}+f{\text{D}}_{\text{W}}.$$

Solving for *f*, we get (9):

(9) $$\left(1-f\right)\Lambda_{11}+f{\text{D}}_{\text{W}}=\lambda_{1}\Rightarrow \Lambda_{11}-f\Lambda_{11}+f{\text{D}}_{\text{W}}=\lambda_{1}f\left({\text{D}}_{\text{W}}-\Lambda_{11}\right)=\lambda_{1}-\Lambda_{11}\Rightarrow f=\frac{\lambda_{1}-\Lambda_{11}}{{\text{D}}_{\text{W}}-\Lambda_{11}},$$

and by extension (10):

(10) $$f=\frac{\lambda_{2}-\Lambda_{22}}{{\text{D}}_{\text{W}}-\Lambda_{22}}\;and\;f=\frac{\lambda_{3}-\Lambda_{33}}{{\text{D}}_{\text{W}}-\Lambda_{33}}.$$

The fraction *f* must be 1 or a proper fraction, and therefore subtracting an equal number from denominator and numerator will decrease the value of the fraction.^1^ Thus (11),

(11) $$\frac{\lambda_{1}}{{\text{D}}_{\text{W}}}\geq \frac{\lambda_{1}-\Lambda_{11}}{{\text{D}}_{\text{W}}-\Lambda_{11}},$$

and we have the following upper bounds on *f* (12):

(12) $$\frac{\lambda_{1}}{{\text{D}}_{\text{W}}}\geq f,\;\frac{\lambda_{2}}{{\text{D}}_{\text{W}}}\geq f,\frac{\lambda_{3}}{{\text{D}}_{\text{W}}}\geq f.$$

Since by definition $\lambda_{1}\geq \lambda_{2}\geq \lambda_{3}$, the lowest upper bound on *f*, termed *f*_*UL*_, is (13):

(13) $$f_{UL}=\frac{\lambda_{3}}{D_{W}}.$$

Considering the physical interpretation of *f*_*UL*_, a characteristic of *λ*_*3*_ is that because the free water component of diffusivity must be equal in all directions, the free water diffusivity cannot be lower than the 3^rd^ eigenvalue. The 3^rd^ eigenvalue *λ*_*3*_ therefore forms an upper limit on the diffusivity due to free water. Assuming that free water contributes proportionally to the measured diffusion tensor, *λ*_*3*_ can be divided by the known diffusivity of water to give an upper limit on the fraction of free water in the voxel.

### 2.3 Water diffusivity as a constant

Water diffusivity is constant for a given pressure and temperature. The body maintains a temperature at approximately 36.7° [16], with small differences between brain and body temperatures under healthy conditions [17]. The brain will be at a similar temperature to the body under normal conditions, with brain trauma or fever leading to variations over a range of up to 1–2°, in the extreme [18].

Intracranial pressure is normally within 7–15 mmHg [19], or ∼1000–2000 Pa. While this variation is high, and may not reflect water pressure in brain tissue, the pressure levels will have very little influence on the water diffusivity, and so the lowest reported values of 1000 Pa were assumed.

Based on a temperature of 310 K, we linearly interpolated between the reported values of 2.30 at 298.15 K and 3.55 at 318.15 K to estimate water diffusivity as 3.04 *10^−9^ m^2^/s (14):

(14) $${\text{D}}_{\text{W}}\left(brain\right)=\;D_{W\left(298.15\text{K}\right)}+\left(310-298.15\right)\times \left(D_{W\left(318.15\text{K}\right)}-D_{W\left(298.15\text{K}\right)}\right)=2.30\times 10^{-9}+\left(310-298.15\right)\times \left(3.55\times 10^{-9}-2.30\times 10^{-9}\right)=3.04\times 10^{-9}$$

Higher true diffusivity due to higher brain temperature would lead to an overestimate of *f*_*UL*_, whereas lower true diffusivity due to cooler brain temperature would lead to an underestimate of *f*_*UL*_. Considering possible extreme conditions over a 2 °C range, diffusivity could vary from 2.92 to 3.17 × 10^−9^ m^2^/s. A more realistic 0.5 °C range, which should easily encompass healthy variation, would lead to a diffusivity range of 3.01–3.07 × 10^−9^ m^2^/s. Since *f*_*UL*_ is an indirect upper limit on the true free water fraction, the errors due to assuming a constant diffusivity of water in the brain should be minimal, even under pathological conditions.

### 2.4 Assumptions

Assumptions include the consistent diffusivity of water, which relates to brain temperature being within a ∼2° range, and that free water contributes proportionally to the diffusivity within a region (voxel). The two-compartment model assumes two independent components that are additive in nature [15], but there are arguments for other models [20]. A further assumption underlying the interpretation of this measure is that the contribution of non-free water compartment is modest. The most likely condition which would challenge this assumption is in dense tissue, with low free water. Thus, lower values of *f*_*UL*_ are at risk of being more divergent from *f* than high values. At the maximal *f* of 1, which will occur in CSF, the estimated fraction *f*_*UL*_ should have good accuracy. Further assumptions relate to the DTI measures, the core of which is that voxel diffusivity is accurately represented by a symmetric tensor calculated from standard single b-value protocol. There is extensive literature addressing alternative representations of diffusivity, for example accounting for non-symmetric diffusivity, non-linear diffusivity across b values, non-Gaussian distribution of water movement, and signal-to-noise and scanning artifacts. Furthermore, several techniques involving customized DTI protocols have been proposed specifically for the calculation of free water [5, 21, 22]. However, for the present method, accounting for such effects was not considered likely to make a substantial difference in *f*_*UL*_ calculation, as the free water measure is intended only as an upper limit to a true measure of *f*.

## 3 Methods

### 3.1 Subjects: IXI dataset

We used the publicly available IXI dataset (http://brain-development.org/ixi-dataset/). This dataset consists of over 500 sets of MRI scans collected from three locations in London (Hammersmith Hospital using a Philips 3T system, Guy's Hospital using a Philips 1.5T system, and Institute of Psychiatry using a GE 1.5T system); full details are provided on the website. We included subjects with T1 and DTI scans, which did not include any from the GE 1.5T, and performed quality checking of the data. The list of included IXI subjects is in Supplementary File 1 (“Supplementary File 1 - IXI subjects.xlsx”). A total of 387 IXI subjects include DTI scans. Referring to the IXI subject numbers, the following were excluded: 555 (inconsistent slice-file sizes); 237, 419, 498, 550 (failure of registration to T1); 324 (partially cut off); and 411, 534, 535, 593, 630, 648, 651 (DTI quality or file corruption issues reflected as incorrect FA/RGB direction color-maps). There were 374 subjects included, as shown in Table 1.

**Table 1:** Subject characteristics; see Supplementary File 1 for subject numbers, and http://brain-development.org/ixi-dataset for individual demographic information.

	All N = 374	Female N = 206	Male N = 168
Age mean ± std [range]	52.1 ± 15.8 [20.1–86.2]	52.1 ± 15.8 [20.1–86.2]	50.0 ± 16.6 [20.2–86.2]
N per scanner (Philips 3T at Hammersmith Hospital or Philips 1.5T at Guy's Hospital)	177 on 3T, 197 on 1.5T	91 on 3T, 115 on 1.5T	86 on 3T, 82 on 1.5T

The full scanning protocols are included at the IXI website (see links at http://brain-development.org/ixi-dataset/). In brief, the T1 images were 0.93 × 0.93 × 1.2 mm resolution with 2 averages, and the DTI images had 15 directions with a b-value of 1000, and resolution of 1.75 × 1.75 × 2.35 mm with 56 slices (3T) or 1.75 × 1.75 × 2.0 mm with 64 slices (1.5T), and 3 averages.

### 3.2 Calculation of *f*_*UL*_

Calculating *f*_*UL*_ is trivial once the eigenvectors of the diffusion tensor have been calculated, as in (15):

(15) $$f_{UL}=\frac{\lambda_{3}}{D_{W\left(318\text{K}\right)}}=\frac{\lambda_{3}}{3.04*10^{-9}}$$

However, since DTI data are often noisy, we set a maximum fraction of 1, and the calculation is Eq. (16):

(16) $$f_{UL}=\text{min}\left(1,\;\frac{\lambda_{3}}{3.04*10^{-9}}\right)$$

This calculation can be performed in most neuroimaging software packages; we used SPM12.

### 3.3 Analysis

We used SPM12 software to analyze the data (http://www.fil.ion.ucl.ac.uk/spm/). The T1-weighted scans were rigid-body coregistered with the MNI template, and manual adjustments were made to correct rotations and shifts. Rotations in particular can interfere with later processing steps. The T1 images were resliced to a common space, and we applied the SPM “Unified segmentation” procedure to bias correct and segment the images [23]. This step creates maps with probabilities of gray matter, white matter and CSF. We passed the segmentation maps to the DARTEL procedure to calculate spatial normalization parameters from native to template space [24]. We used the VBM8 template that is available with SPM, and is coregistered with MNI space (http://www.neuro.uni-jena.de/vbm/).

We used the SPM Diffusion II toolbox for processing the DTI data (https://sourceforge.net/projects/spmtools/). Each series were used to calculate the diffusion tensor D. The three eigenvalues of the tensor were calculated with Eigen decomposition, and *f*_*UL*_ was calculated according to (16). We included an SPM12 batch file (Supplementary File 2: “Supplementary File 2 - spmjob_fwcalc.txt”) implementing this calculation using the “im_calc” tool, which takes as input the third eigenvalue image. We used the b0 images for a two-step process to accurately register the DTI scans into a common space [see Appendix A in 25]. For each subject, the b0 image was coregistered to the preprocessed T1 in native space using a warping procedure to allow for distortions inherent in the DTI scans. In brief, this step involves using the segmented tissue types from the T1 unified segmentation as priors in the DTI unified segmentation [25]. The result is a mapping from DTI to T1 space, which is applied to the calculated indices including *f*_*UL*_. The second step is to apply the T1 DARTEL normalization parameters to the indices, resulting in spatially normalized maps that can be combined or compared across subjects on a voxel-by-voxel basis.

Values from regions of interest from the atlas provided with SPM12 were extracted with the SPM Volumes utility. The tissue labels were derived from the “MICCAI 2012 Grand Challenge and Workshop on Multi-Atlas Labeling” (https://masi.vuse.vanderbilt.edu/workshop2012/index.php/Challenge_Details), based MRI scans from the OASIS project (http://www.oasis-brains.org/), and the labeled data were provided by Neuromorphometrics, Inc. under academic subscription (http://neuromorphometrics.com/). We also extracted values from the entire intracranial space by using the TIV, defined as regions where the sum of CSF, gray and white matter probabilities was greater than 0.5. Values of *f*_*UL*_ and other DTI indices, including mean diffusivity (MD), axial diffusivity and radial diffusivity were extracted from each voxel in each region in each subject. Similarly, values of the probabilities of CSF, gray and white matter were extract from the *f*_*UL*_ voxels, using linear interpolation to sample the T1-derived values from voxels DTI space (since T1 images were in a different space and with different voxel sizes than the DTI images). Descriptive statistics and distributions of each measure were calculated and displayed using MATLAB functions. Correlations between *f*_*UL*_ and other measures were performed with MATLAB. Scatterplots of measures versus *f*_*UL*_ were created with MATLAB, with transparent markers due to the large number of points (95% transparency). TIV values were subsampled such that a subset of fewer than 1,000,000 points were included in any one scatterplot.

## 4 Results

**Fig. 4:**
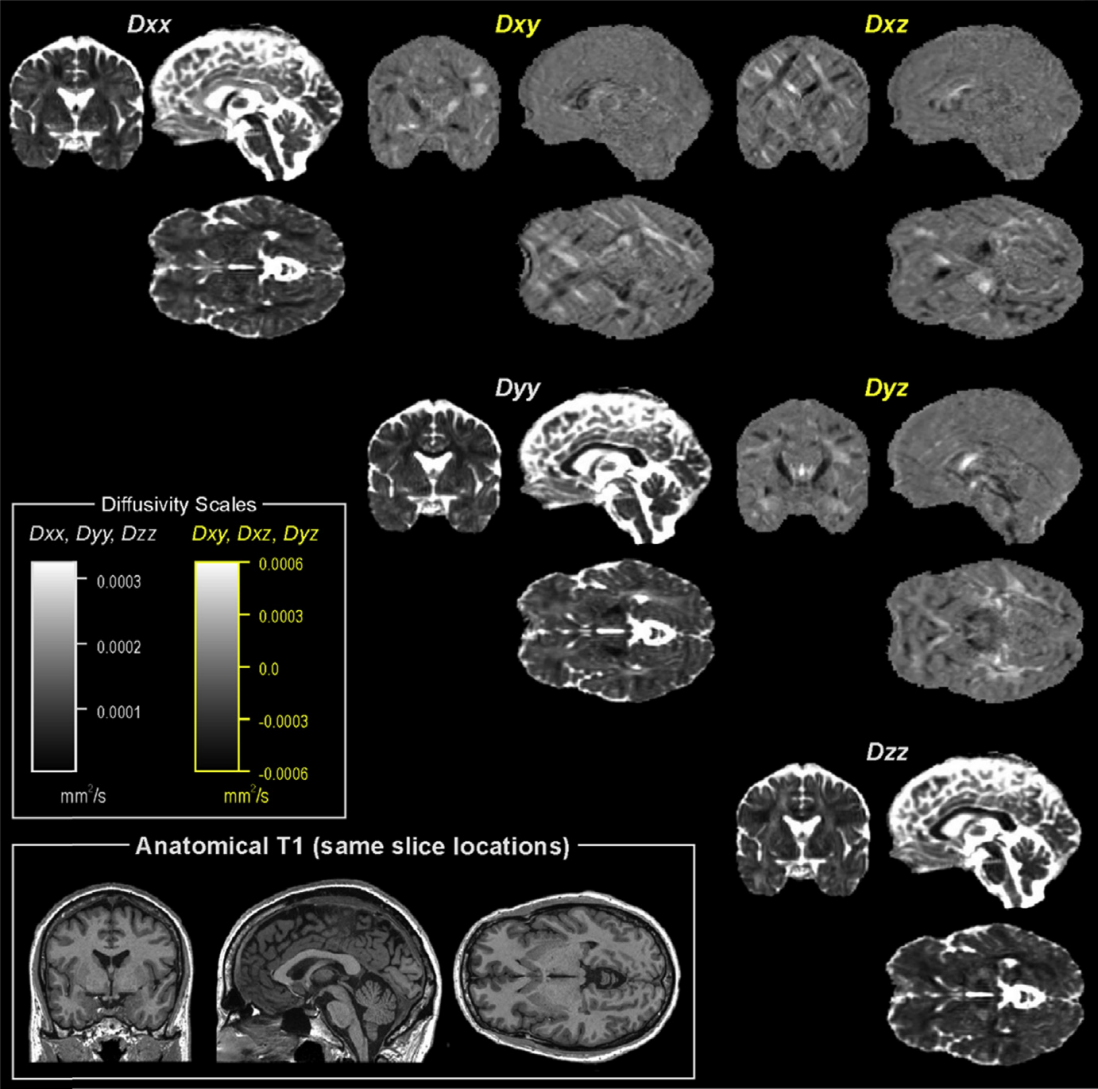
Example of diffusion tensor diffusivity terms for a single subject. Diagonal and non-diagonal terms are shown on different scales to best illustrate structure.

**Fig. 5:**
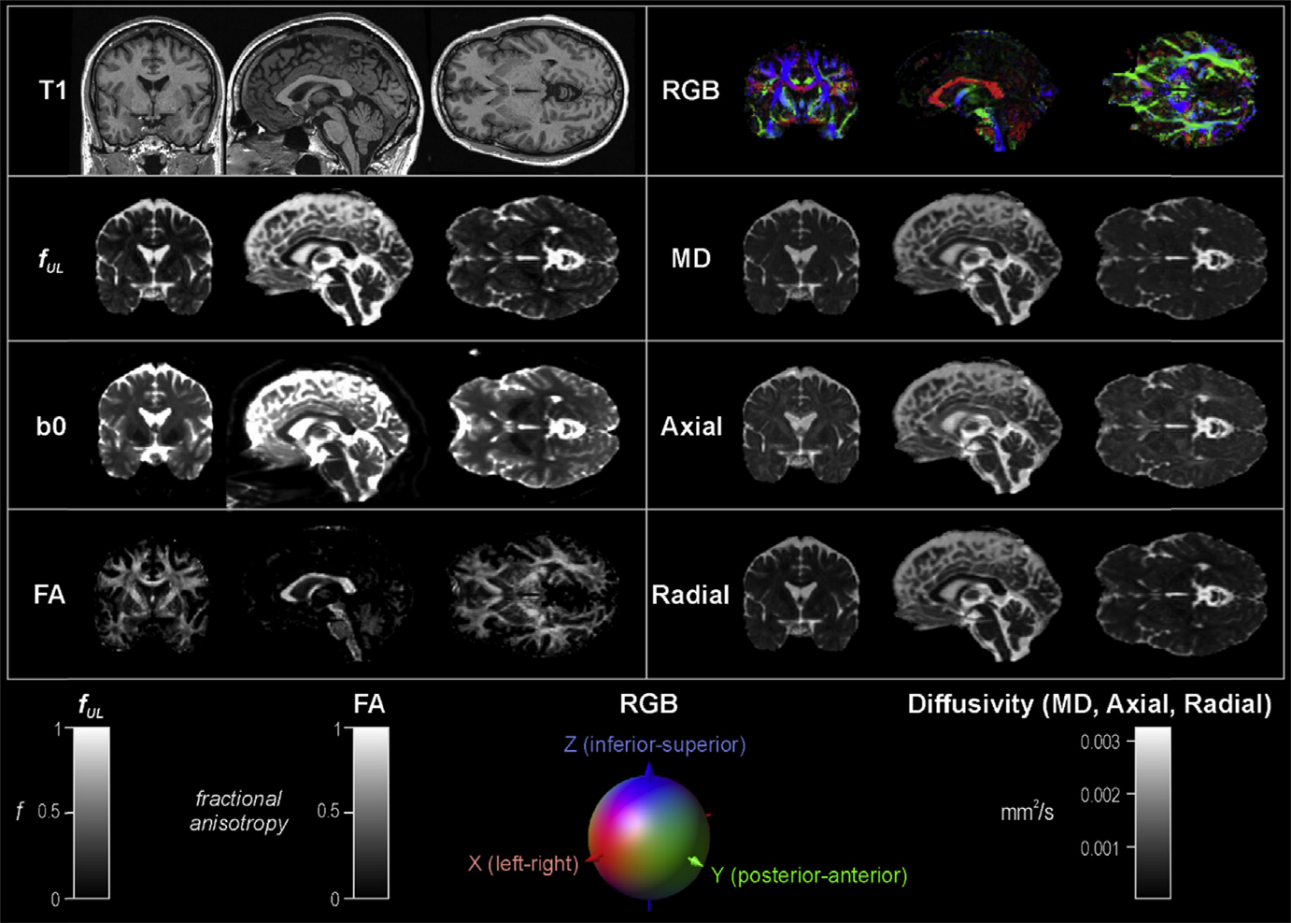
Example of DTI-based anatomy and indices for a single subject. T1: T1-weighted anatomical reference; all DTI-based images are in T1 space. *f*_*UL*_: free water fraction upper limit. b0: b0 image. FA: fractional anisotropy. RGB: color-coded directional anatomy, as per scale. MD: mean diffusivity. Axial: axial diffusivity. Radial: radial diffusivity. Scales for indices are at bottom.

We select the subject closest to the mean age of our sample, a male age 42.6 years, BMI 29.4 kg/m^2^, and right handed. The maps of the tensor **D** components are shown in Fig. 4. Other DTI images and indices are shown in Fig. 5, including the standard FA and diffusivities (mean, axial and radial), and FW.

**Fig. 6:**
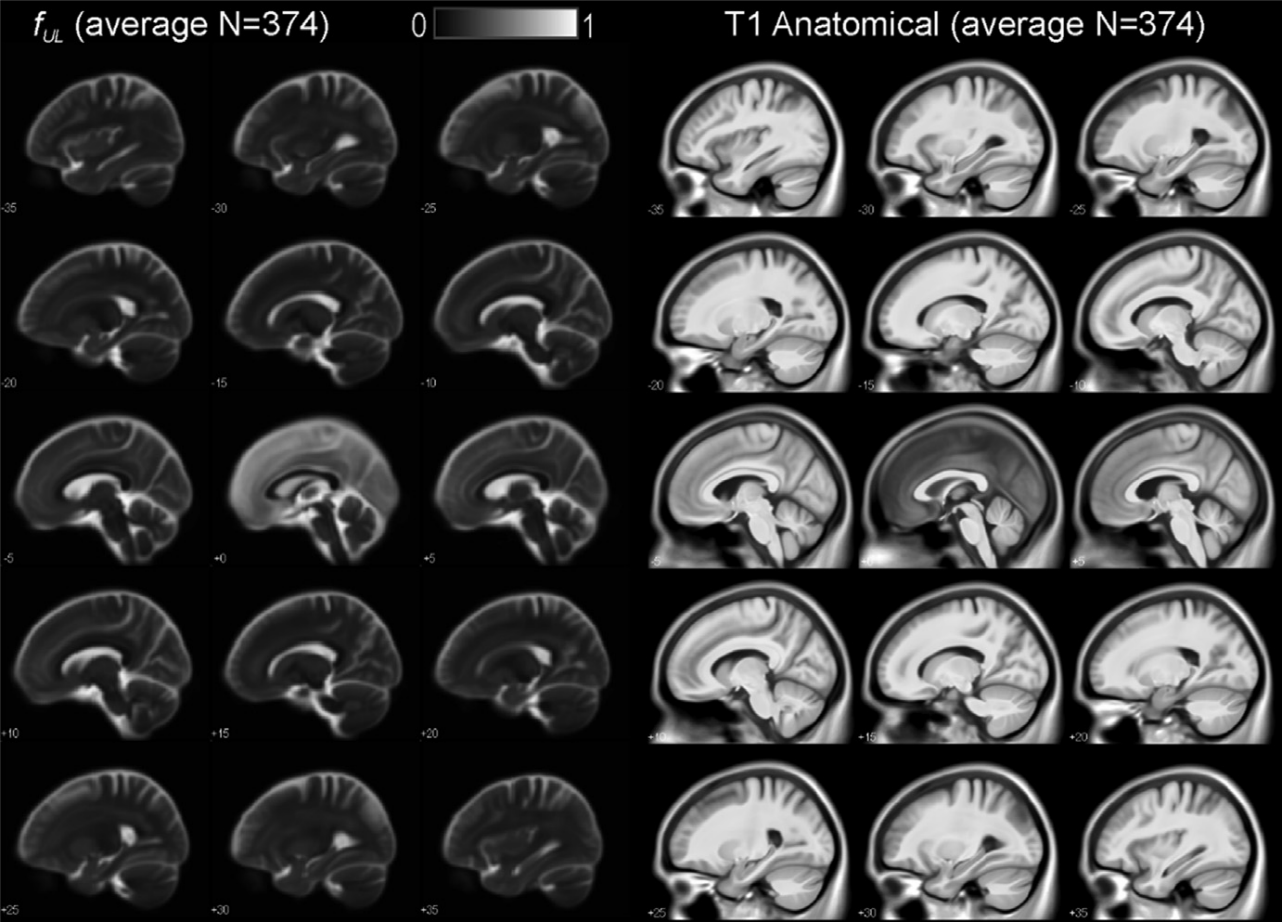
Average *f*_*UL*_ free water fraction upper limit and corresponding anatomical T1-weighted scans for 375 IXI subjects. Locations of sagittal slices are in x-values in MNI space.

Fig. 6 illustrates the average normalized *f*_*UL*_ maps over the 374 subjects. The average T1 anatomical is included for reference on the right of Fig. 6.

The mean and standard deviation of *f*_*UL*_, T1-derived tissue probability maps, and other DTI indices are shown in Table 2 for 14 selected bilateral VOIs and three tissue compartments. The values for the full set of 136 VOIs are in Supplementary File 3 (“Supplementary File 3 - Correlations with FW by modality.xlsx”). As expected, the highest *f*_*UL*_ values were in regions with high probabilities of CSF. Values in gray matter compartment were slightly higher than the white matter compartment, but the lowest values were in the pallidum, which contains mixed gray and white tissue.

**Table 2:** Values by region of tissue probability (gray and white matter and CSF) and DTI-derived indices; mean ± std over 374 subjects. Number of voxels of region are based on a voxel size of 1.5 mm^3^.

	N voxels	Gray Matter	White Matter	CSF	MD	axial	FA	FW	radial
CSF	117049	0.1321 ± 0.2485	0.0044 ± 0.0423	0.7580 ± 0.2926	0.0018 ± 0.0009	0.0022 ± 0.0011	0.2046 ± 0.1257	0.4511 ± 0.2221	0.0015 ± 0.0008
Gray matter	256549	0.7724 ± 0.2988	0.1024 ± 0.2260	0.1206 ± 0.2451	0.0009 ± 0.0004	0.0011 ± 0.0004	0.2208 ± 0.1053	0.2302 ± 0.1050	0.0008 ± 0.0003
White matter	162773	0.1119 ± 0.2301	0.8808 ± 0.2417	0.0055 ± 0.0483	0.0007 ± 0.0002	0.0010 ± 0.0003	0.4226 ± 0.1441	0.1397 ± 0.0519	0.0005 ± 0.0002
Left Hippocampus	1359	0.8438 ± 0.2672	0.0368 ± 0.1349	0.1176 ± 0.2425	0.0011 ± 0.0004	0.0013 ± 0.0005	0.2265 ± 0.0919	0.2734 ± 0.1055	0.0009 ± 0.0003
Right Hippocampus	1457	0.8541 ± 0.2610	0.0287 ± 0.1135	0.1153 ± 0.2435	0.0011 ± 0.0004	0.0013 ± 0.0005	0.2248 ± 0.0895	0.2734 ± 0.1079	0.0009 ± 0.0004
Left MOG middle occipital gyrus	2659	0.6734 ± 0.3550	0.1602 ± 0.2997	0.1601 ± 0.2873	0.0010 ± 0.0004	0.0012 ± 0.0005	0.2101 ± 0.1020	0.2562 ± 0.1217	0.0009 ± 0.0004
Right MOG middle occipital gyrus	2263	0.6738 ± 0.3535	0.1454 ± 0.2868	0.1650 ± 0.2829	0.0010 ± 0.0004	0.0012 ± 0.0005	0.2145 ± 0.0964	0.2514 ± 0.1085	0.0009 ± 0.0003
Left PCgG posterior cingulate gyrus	1877	0.6969 ± 0.3493	0.0877 ± 0.2133	0.2120 ± 0.3323	0.0009 ± 0.0004	0.0011 ± 0.0004	0.2052 ± 0.0952	0.2551 ± 0.1095	0.0009 ± 0.0003
Right PCgG posterior cingulate gyrus	1648	0.7383 ± 0.3210	0.1020 ± 0.2264	0.1571 ± 0.2851	0.0009 ± 0.0003	0.0011 ± 0.0004	0.2109 ± 0.0925	0.2411 ± 0.1024	0.0008 ± 0.0003
Left Cerebellum White Matter	4948	0.1392 ± 0.2664	0.8237 ± 0.3130	0.0351 ± 0.1613	0.0007 ± 0.0004	0.0011 ± 0.0005	0.4411 ± 0.1507	0.1454 ± 0.1007	0.0006 ± 0.0003
Right Cerebellum White Matter	4847	0.1114 ± 0.2261	0.8635 ± 0.2663	0.0232 ± 0.1201	0.0007 ± 0.0003	0.0011 ± 0.0004	0.4468 ± 0.1464	0.1409 ± 0.0853	0.0005 ± 0.0003
Left SPL superior parietal lobule	5225	0.4955 ± 0.3931	0.1335 ± 0.2805	0.3621 ± 0.4095	0.0012 ± 0.0006	0.0014 ± 0.0006	0.1998 ± 0.1109	0.3220 ± 0.1774	0.0011 ± 0.0006
Right SPL superior parietal lobule	5249	0.4720 ± 0.3950	0.1138 ± 0.2588	0.4024 ± 0.4178	0.0012 ± 0.0006	0.0014 ± 0.0006	0.1946 ± 0.1034	0.3375 ± 0.1779	0.0011 ± 0.0006
Left Pallidum	559	0.0347 ± 0.0905	0.9609 ± 0.0963	0.0012 ± 0.0059	0.0007 ± 0.0001	0.0010 ± 0.0001	0.4009 ± 0.1427	0.1375 ± 0.0397	0.0005 ± 0.0001
Right Pallidum	564	0.0253 ± 0.0744	0.9700 ± 0.0816	0.0014 ± 0.0064	0.0007 ± 0.0001	0.0010 ± 0.0001	0.4259 ± 0.1499	0.1334 ± 0.0401	0.0005 ± 0.0001
Left PP planum polare	893	0.6095 ± 0.3765	0.0095 ± 0.0515	0.3775 ± 0.3801	0.0012 ± 0.0005	0.0014 ± 0.0006	0.1715 ± 0.0639	0.3357 ± 0.1390	0.0011 ± 0.0005
Right PP planum polare	783	0.5859 ± 0.3846	0.0048 ± 0.0288	0.4054 ± 0.3861	0.0013 ± 0.0006	0.0015 ± 0.0007	0.1688 ± 0.0584	0.3628 ± 0.1516	0.0012 ± 0.0005

Correlations between *f*_*UL*_ and tissue probabilities, and with other DTI indices for each VOI are shown in Table 3. The radial diffusivity was most strongly correlated with *f*_*UL*_, with average coefficients ranging from over 0.99 to 0.92. FA showed mostly significant negative correlations. Only moderate correlations with CSF likely relate to the lack of variation in CSF probability or *f*_*UL*_ for voxels encompassing CSF. The full correlation table for 136 VOIs is in Supplementary File 4 (“Supplementary File 4 - Description by modality.xlsx”).

**Table 3:** Correlations with f_UL_ of tissue probability and DTI-derived indices in regions. The percentages of the voxels in the VOI that were included for the calculation are shown (number of voxels shown in Table 2). Significant correlations (P < 0.05) indicated by a “*”.

	Gray Matter		White Matter		CSF		MD		axial		FA		radial	
	Corr (r)	N (%)	Corr (r)	N (%)	Corr (r)	N (%)	Corr (r)	N (%)	Corr (r)	N (%)	Corr (r)	N (%)	Corr (r)	N (%)
CSF	−0.32	92%	−0.11	68%	*0.42	100%	*0.94	100%	*0.82	100%	−0.25	100%	*0.98	100%
Gray matter	−0.23	100%	−0.23	99%	*0.49	100%	*0.94	100%	*0.79	100%	*−0.54	100%	*0.98	100%
White matter	0.24	100%	−0.28	100%	0.22	91%	0*.80	100%	*0.37	100%	*−0.69	100%	*0.92	100%
Left Hippocampus	−0.35	100%	−0.06	99%	*0.41	100%	*0.94	100%	*0.80	100%	*−0.41	100%	*0.98	100%
Right Hippocampus	−0.37	100%	−0.04	100%	*0.42	100%	*0.93	100%	*0.80	100%	*−0.41	100%	*0.98	100%
Left MOG middle occipital gyrus	*−0.26	100%	*−0.37	100%	*0.70	100%	*0.96	100%	*0.83	100%	−*0.60	100%	*0.99	100%
Right MOG middle occipital gyrus	−0.28	100%	−0.33	99%	*0.69	100%	*0.93	100%	*0.77	100%	*−0.61	100%	*0.98	100%
Left PCgG posterior cingulate gyrus	*−0.27	100%	−0.21	87%	*0.47	100%	*0.93	100%	*0.77	100%	*−0.58	100%	*0.98	100%
Right PCgG posterior cingulate gyrus	*−0.24	100%	−0.22	95%	*0.48	100%	*0.93	100%	*0.76	100%	*−0.61	100%	*0.98	100%
Left Cerebellum White Matter	0.05	100%	−0.07	100%	0.05	84%	*0.82	100%	*0.51	100%	*−0.54	100%	*0.9249	100%
Right Cerebellum White Matter	0.05	100%	−0.07	100%	0.05	82%	*0.81	100%	*0.49	100%	*−0.55	100%	*0.92	100%
Left SPL superior parietal lobule	*−0.43	100%	*−0.37	100%	*0.75	100%	*0.98	100%	*0.90	100%	*−0.64	100%	*0.9940	100%
Right SPL superior parietal lobule	*−0.47	100%	*−0.36	100%	*0.76	100%	*0.97	100%	*0.89	100%	*−0.64	100%	*0.9930	100%
Left Pallidum	0.19	100%	−0.19	100%	0.11	89%	*0.79	100%	0.28	100%	*−0.78	100%	*0.9349	100%
Right Pallidum	0.17	100%	−0.17	100%	0.07	99%	*0.78	100%	0.21	100%	*−0.80	100%	*0.9270	100%
Left PP planum polare	*−0.46	100%	−0.04	95%	*0.49	100%	*0.96	100%	*0.87	100%	−0.25	100%	*0.9819	100%
Right PP planum polare	*−0.49	100%	−0.02	98%	*0.50	100%	*0.95	100%	*0.87	100%	−0.22	100%	*0.9802	100%

**Fig. 7:**
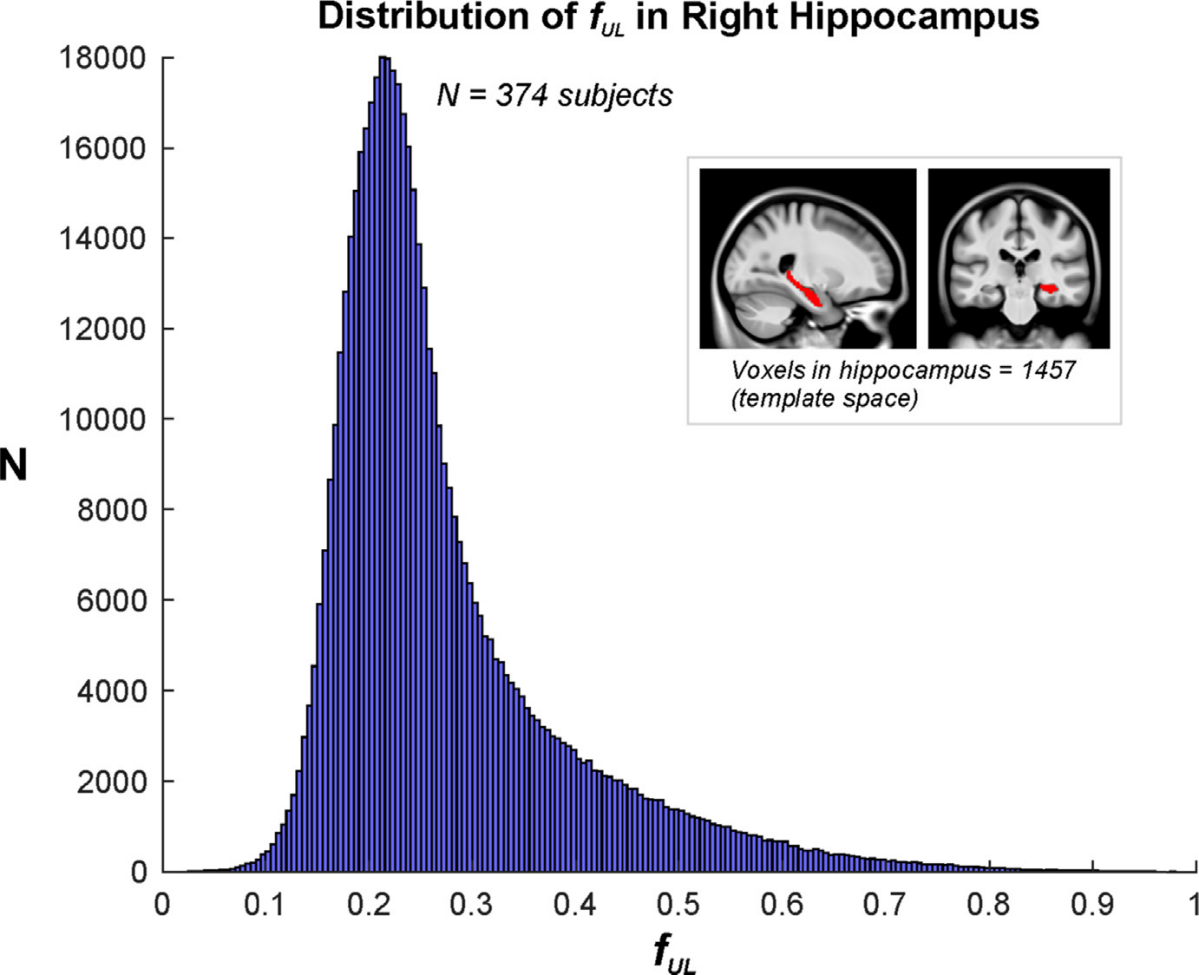
Distribution of *f*_*UL*_ values in hippocampus of 374 IXI subjects. Insert illustrates location of hippocampus VOI in template space.

In addition to a range of *f*_*UL*_ values between brain regions and between overall tissue types, there was also variation in *f*_*UL*_ values within a particular VOI. For example, the distribution of *f*_*UL*_ values in the hippocampus is shown in Fig. 7, derived from the entire hippocampi in 374 subjects. The majority of the values lie between 0.1 and 0.6. This structure is a combination of gray and white matter, and in some subjects with atrophy CSF is also present in the hippocampal region.

**Fig. 8:**
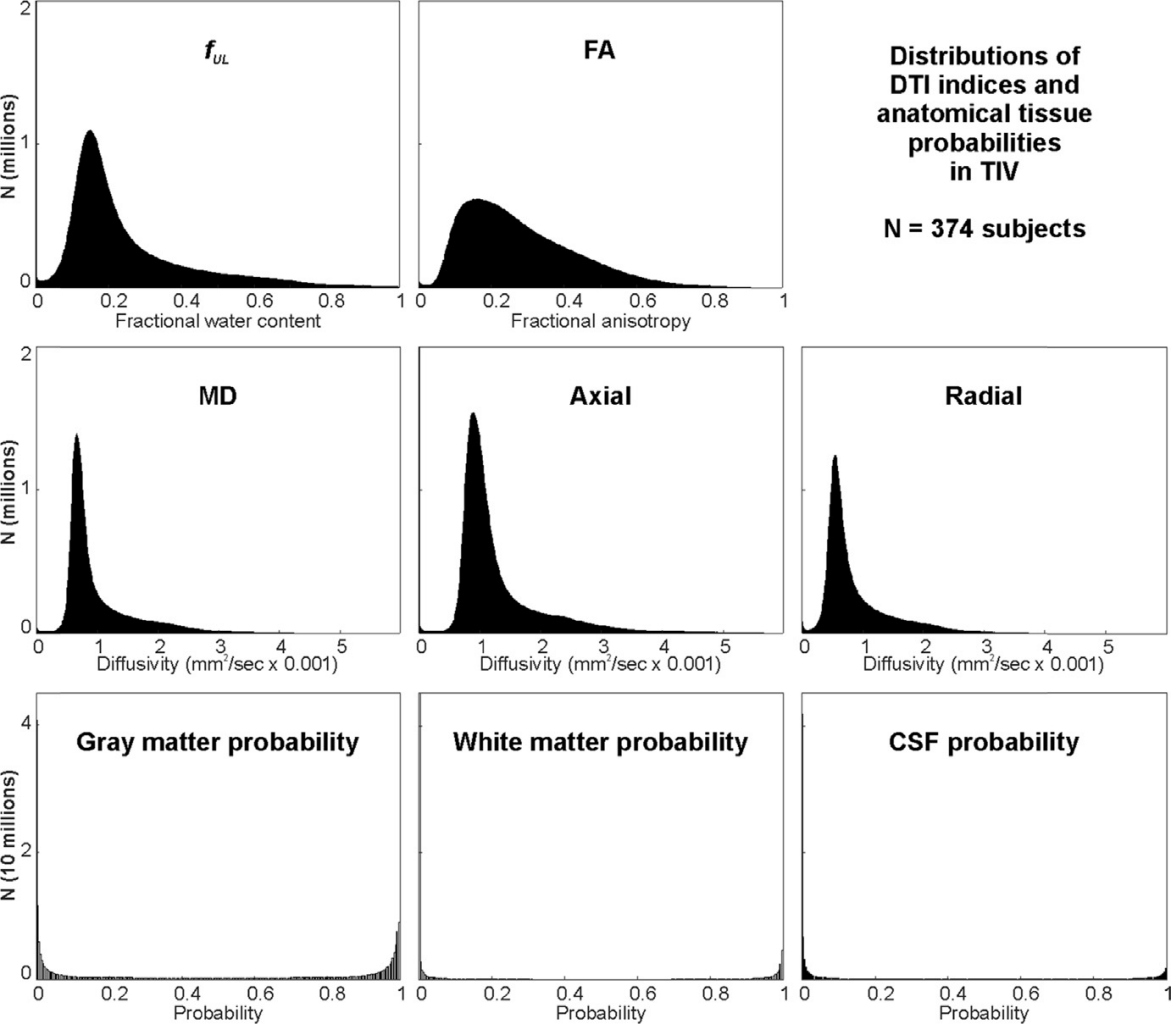
Distributions of indices for all voxels in intracranial space (TIV) in 374 IXI subjects. *f*_*UL*_: free water fraction upper limit; FA: fractional anisotropy; MD: mean diffusivity; Axial: axial diffusivity; Radial: radial diffusivity. Tissue probabilities are based on SPM12 tissue segmentation. Y-axes are unscaled, and represent raw counts.

The final analyses were performed using all voxels within the entire intracranial volume (TIV). The values from 511,190 TIV voxels in each of the 374 subjects were extracted. Table 4 presents the mean and standard deviation for tissue probabilities and DTI-based indices, as well as the correlation statistics with respect to *f*_*UL*_. *f*_*UL*_ correlated significantly with radial and mean diffusivity, and to a less significant extent with axial diffusivity. CSF probability showed only a non-significant trend towards a correlation. FA showed a significant albeit not strong negative relationship with *f*_*UL*_. The distribution of *f*_*UL*_ across TIV in Fig. 8 illustrates a slightly wider range of values than just in the hippocampus (Fig. 7). For comparison, the distributions of other DTI indices and tissue probabilities are also shown. Note that the probabilities of gray and white matter and CSF probabilities are distributed closely to either 0 or 1, whereas the DTI-based indices show a wider spread.

**Table 4:** Descriptive and correlation relative to f_UL_ statistics of indices for 374 IXI subjects in entire TIV space (511,190 voxels). For T1-based tissue probabilities, some voxels in some subjects were excluded if the T1-based value was outside the brain; the N column reflects the percent voxels included.

	Descriptive		Pearson's correlation with *f*_*UL*_	*P*	N (%)
	mean	std	r		
*f*_*UL*_	0.251	±0.173	-	-	-
Gray matter probability	0.431	±0.421	−0.10	0.085	98%
White matter probability	0.314	±0.425	−0.23	0.13	93%
CSF probability	0.227	±0.364	0.40	0.067	97%
FA	0.278	±0.156	−0.52	0.018	100%
MD	0.001014	±0.000621 mm/s^2^	0.90	<0.001	100%
Axial	0.001300	±0.000762 mm/s^2^	0.67	0.013	100%
Radial	0.000871	±0.000571 mm/s^2^	0.96	<0.001	100%

**Fig. 9:**
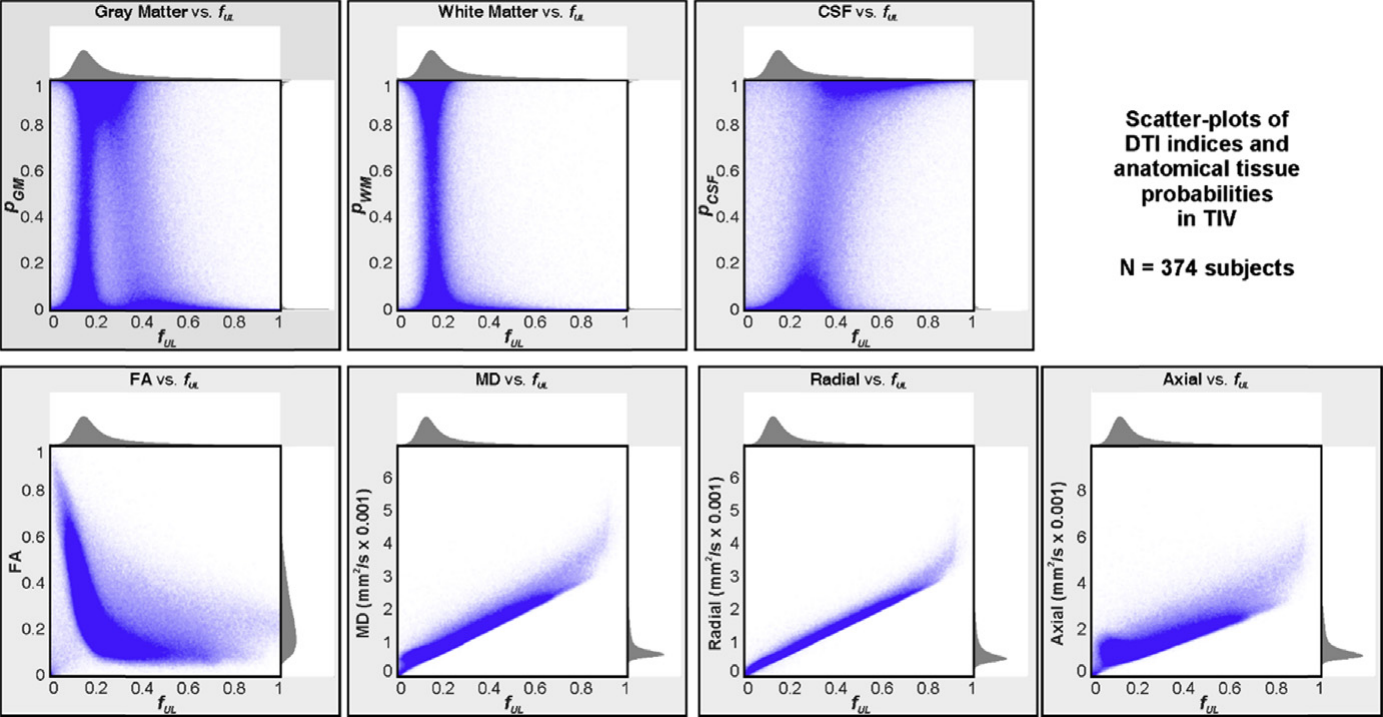
Scatterplots of indices with respect to *f*_*UL*_ for all voxels in intracranial space (TIV) in 374 IXI subjects. Each plot was subsampled from the original 200 million points to ∼1 million; each point is 95% translucent, effectively resulting in a density plot. *f*_*UL*_: free water fraction upper limit; FA: fractional anisotropy; MD: mean diffusivity; Axial: axial diffusivity; Radial: radial diffusivity. Tissue probabilities are based on SPM12 tissue segmentation. The distributions of each measure are shown above and right of the scatter plot.

The relationships between *f*_*UL*_ and other measures are shown in scatter plots in Fig. 9. Each DTI index and tissue probability is plotted against *f*_*UL*_ for TIV voxels across the 374 subjects. Because of the large number of points (∼200 million), only every 200^th^ point was plotted, such that each graph contains approximately 1 million values. Diffusivity indices showed positive relationships with *f*_*UL*_, with radial being most closely correlated. FA showed an inverse hyperbolic relationship with *f*_*UL*_. The gray and white matter tissue probabilities showed little relationship with *f*_*UL*_, and CSF probability exhibited a modest positive relationship.

## 5 Discussion

The measure of an upper limit to the fraction of free water *f*_*UL*_ is a DTI-based index indirectly related to voxel free water content. The measure provides an upper limit on the free water fraction *f* in the context of a two-compartment model consisting of tissue and free water. In the intracranial space, the measure is distribution across the range from 0 to 1, with most voxels falling in the 0.1 to 0.6 fraction. The measure is most closely related to radial diffusivity, followed by axial and MD, and negatively related to FA.

The *f*_*UL*_ measure is more closely related to other DTI indices than tissue probabilities. Although *f*_*UL*_ is related to the probability of a voxel containing CSF, the correlation is weak, suggesting *f*_*UL*_ is measuring a characteristic of mixed “tissue,” as expected, as opposed to a marker of CSF-containing voxels. There is little relationship between *f*_*UL*_ and probabilities of gray or white matter, which likely is due to the close to categorical nature of those probabilities.

The assumptions underlying the interpretation of the measure include accurate calculation of the diffusion tensor and little variation in water diffusivity due to temperature changes. Furthermore, the two compartment model assumes “fluid” and tissue components of a particular voxel, with “fluid” behaving like water. These assumptions are all likely violated to some degree. Based on the calculations with Eq. (3), temperature is unlikely to cause substantial error. However, noise and distortion in the DTI data is likely contributing both random and systematic error to the tensor calculation [26, 27, 28, 29]. A reflection of such error could be the occasional occurrence of voxels with *f*_*UL*_ greater than 1, which is theoretically impossible were the assumptions correct. The impact of variations in the nature of intercellular fluid is unknown, but such influences may affect the measure.

In conclusion, we presented the theory and implementation of a measure of an upper limit to the fraction of free water *f*_*UL*_ within a voxel, based on scaling the third eigenvalue from the DTI-derived diffusion tensor. The measure is calculated from a single tensor model, which can be derived from a single shell at one b-value. The values of *f*_*UL*_ are closely related but not identical to radial diffusivity. The measure may be useful as a starting point in calculating the true free water fraction *f* of a voxel, or as an easy-to-calculate tissue characteristic indirectly related to *f*.

## Declarations

### Author contribution statement

Paul M. Macey: Conceived and designed the experiments; Performed the experiments; Analyzed and interpreted the data; Contributed reagents, materials, analysis tools or data; Wrote the paper.

M. Albert Thomas, Luke A. Henderson: Analyzed and interpreted the data; Wrote the paper.

### Funding statement

This work was supported by the National Institutes of Health (NR-01693).

### Competing interest statement

The authors declare no conflict of interest.

### Additional information

Data associated with this study can be found at http://brain-development.org/ixi-dataset.

## Appendix A Supplementary data

Supplementary File 1 - IXI subjects Supplementary File 1 - IXI subjects

Supplementary File 2 - spmjob_fwcalc Supplementary File 2 - spmjob_fwcalc

Supplementary File 3 - Correlations with FW by modality Supplementary File 3 - Correlations with FW by modality

Supplementary File 4 - Description by modality Supplementary File 4 - Description by modality

## Footnotes

1 Proof: consider a fraction *f* formed with two positive numbers *n* and *d* (*f* = *n*/*d*), and a second fraction *g* based on subtracting a positive number *p* from numerator and denominator of *f* (*g* = (*n* − *p*)/(*d* − *p*)). Solving for n in both fractions leads to *d*(*f* − *g)* = p(1 − g). The sign must be the same on both sides, so if g > 1 the right side is negative and f − g < 0; similarly, if g = 1 then f = g, and (in our case) if g < 1 then f − g > 0.
